# Survey Regarding Gastrointestinal Stoma Construction and Closure in Japan

**DOI:** 10.1002/ags3.12521

**Published:** 2021-11-06

**Authors:** Yoshiko Ando, Arata Takahashi, Makoto Fujii, Hiroshi Hasegawa, Toshimoto Kimura, Hiroyuki Yamamoto, Tetsuya Tajima, Yukio Nishiguchi, Yoshihiro Kakeji, Hiroaki Miyata, Yuko Kitagawa

**Affiliations:** ^1^ Department of Nursing Japanese Red Cross Osaka Hospital Osaka Japan; ^2^ Department of Health Science Graduate School of Medicine Osaka University Osaka Japan; ^3^ NCD Data Quality Management Subcommittee The Japanese Society of Gastroenterological Surgery Tokyo Japan; ^4^ Department of Health Policy and Management School of Medicine Keio University Tokyo Japan; ^5^ Department of Healthcare Quality Assessment Graduate School of Medicine The University of Tokyo Tokyo Japan; ^6^ Project Management Subcommittee The Japanese Society of Gastroenterological Surgery Tokyo Japan; ^7^ Department of Surgery Osaka City Juso Hospital Osaka Japan; ^8^ Database Committee The Japanese Society of Gastroenterological Surgery Tokyo Japan; ^9^ The Japanese Society of Gastroenterological Surgery Tokyo Japan

**Keywords:** colostomy, enterostomy, gastrointestinal stoma closure, gastrointestinal stoma construction, national clinical database, ostomy surgery

## Abstract

**Background and Aim:**

In Japan, the actual number of stoma constructions and stoma closures is not known. The aim of this study was to conduct a survey to determine the number of gastrointestinal stoma constructions and closures in Japan.

**Methods:**

Enrolled participants comprised patients undergoing selected gastrointestinal surgeries who were recorded in the National Clinical Database. This database uses the “Common Items for Gastrointestinal Surgeons.” These procedures were formulated by the Japanese Society of Gastroenterological Surgery during 2013–2018.

**Results:**

According to the National Clinical Database, a total of 154,323 gastrointestinal stomas were constructed between January 1, 2013 and December 31, 2018. By procedure, there were 78,723 cases of stoma construction, 39,653 of abdominoperineal resection, 2470 total pelvic exenteration procedures, and 33,572 Hartmann's procedures. The ratio of stoma closures to stoma constructions increased annually in patients under 70 y of age but not in older patients. Approximately 35% of total colectomies, 60% of proctocolectomies, and 20% of low anterior resections were accompanied by stoma construction. The number of patients with rectal cancer who underwent colostomy increased gradually during the study period and the number who underwent stoma construction increased among older patients.

**Conclusion:**

The number of cases of gastrointestinal stoma construction has increased gradually in Japan, and the proportion of older patients is increasing each year. The purposes and surgical techniques for stoma construction are diverse and are expected to increase in Japan, a super‐aged society.

## Graphical abstract

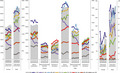

## 1 INTRODUCTION

Ostomates need to learn new skills after surgery and overcome many challenges so as to return to a normal social life, because defecation management after the construction of a gastrointestinal stoma requires special skills.^1^, ^2^, ^3^ In Japan, it is estimated that there are currently approximately 210,000 people who have received physical disability certificates, which can be applied for after the installation of a permanent stoma.^4^ However, the number of temporary stomas and the number of stoma closures cannot be ascertained in the same way, and there are no data available to clearly ascertain the number of new gastrointestinal stomas and the number of people who have them. Additionally, patient background information, such as basic information and surgical information, has not been collected. Thus, it is difficult to grasp the actual situation regarding the number of constructed stomas and stoma closures, together with the patient backgrounds, according to the current official statistics. It is therefore unclear how many ostomates actually exist in Japan at present and the background under which their stoma was constructed. To plan specific support measures for ostomates in Japan, which is a super‐aged society unparalleled in the world in terms of its medical care technology and social security system,^5^ it is necessary to understand the trends in the construction and closure of gastrointestinal stomas and the current status of the background of gastrointestinal stoma construction.

The National Clinical Database (NCD) of Japan, which began registering data in 2011, is a large nationwide database covering more than 95% of the surgeries performed by general surgeons in Japan. At the end of January in 2021, 5404 facilities have enrolled in the NCD and approximately 1,500,000 cases are registered every year.^6^, ^7^ In a validation study using 2016 data conducted by the gastroenterological section of the NCD, the Japanese Society of Gastroenterological Surgery (JSGS), patient demographics, surgical outcomes, and processes were proven to be highly institutionalized.^8^, ^9^ Therefore, by extracting cases of gastrointestinal stoma construction and closure from the NCD database, the number of gastrointestinal stomas constructed and their co‐procedures, the percentage of stomas constructed among them, and the patient background including age group and preoperative information, can be used to confirm the actual situation of gastrointestinal stoma construction in Japan. At the same time, the number of gastrointestinal stoma closures can serve as a valuable dataset to predict the number of people with temporary stomas. In the present study, we conducted a survey regarding the number of gastrointestinal stoma constructions and closures in Japan.

## 2 PARTICIPANTS AND METHODS

The enrolled participants were patients who underwent selected gastrointestinal surgical procedures and who had surgical data recorded in the NCD. The NCD uses the “Common Items for Gastrointestinal Surgeons” as defined in the “Training Curriculum for Board Certified Surgeons in Gastroenterology.” These procedures were formulated by the JSGS. The study period covered 2013–2018, and the data were extracted according to the conditions related to the construction of gastrointestinal stoma.

### 2.1 Surgical procedure

The total number of cases of gastrointestinal stoma was classified into four categories: abdominoperineal resection (APR), total pelvic exenteration (TPE), Hartmann's procedure, and stoma construction. Stoma construction includes enterostomy and colostomy. To exclude “colostomy” as a route for nutritional injection, cases of concurrent gastric and esophageal surgery were excluded. The total number of stomas was defined as the total number of stomas in the four categories.

For enterostomy and colostomy, those associated with esophageal and gastric surgeries were excluded. In cases of total colectomy, proctocolectomy and ileoanal anastomosis, and low anterior resection (LAR), patients without a record of a concomitant procedure were counted separately from stoma nonconstruction. Stoma closure included enterostoma closure, colostoma closure, and stoma closure.

### 2.2 Statistical analysis

Descriptive statistics were conducted for the number of procedures performed, by sex and age group, during 2013–2018 for stoma construction (except for APR, TPE, and Hartmann's procedure), APR, TPE, and Hartmann's procedure. The total number of cases of stoma construction and stoma closure was also analyzed by sex and age group over time. Additionally, the number of patients with and without stoma construction, patients' sex, and patients' age group were counted for the three techniques of total colectomy, proctocolectomy and ileoanal anastomosis, and LAR, and changes over time were examined. In malignant neoplasms of the rectum (International Classification of Diseases, Tenth Revision code: C20), the following characteristics were compared: age at surgery, sex, year of surgery, preoperative chemotherapy within 30 d, preoperative chemotherapy within 90 d, preoperative radiotherapy within 90 d before surgery, American Society of Anesthesiologists physical status (ASA‐PS) classification at surgery, Union for International Cancer Control tumor–node–metastasis (UICC TNM) classification, emergency surgery, endoscopic surgery, and characteristics of cancer remnants in the five procedures (LAR with or without diverting stoma, APR, Hartmann's procedure, stoma construction). Additionally, malignant neoplasm and benign tumors were divided into two groups: LAR with or without stoma, age at surgery, sex, year of surgery, presence of diabetes (diet therapy only, oral agents, insulin treatment, no treatment), preoperative presence of dyspnea within 30 d, preoperative ADL within 30 d, chronic obstructive pulmonary disease, preoperative presence of dialysis within 14 d, immediately preoperative highly advanced cancer with multiple metastases, long‐term steroid treatment, ASA‐PS at surgery, classification at the time of surgery, emergency surgery, presence of endoscopic surgery, preoperative chemotherapy within 30 d and within 90 d, and preoperative radiotherapy within 90 d. For malignant neoplasm, UICC TNM classification and stage were also used as comparison items. All descriptive statistics were performed using IBM SPSS v. 26 (IBM, Armonk NY).

## 3 RESULTS

### 3.1 Annual changes in stoma construction surgeries by sex and age group

A total of 154,323 gastrointestinal stomas were surgically constructed and reported in the NCD between January 1, 2013 and December 31, 2018. By procedure, there were 78,723 stoma constructions (except for APR, TPE, and Hartmann's procedure), 39,653 APRs, 2470 TPEs, and 33,572 Hartmann's procedures.

**FIGURE 1:**
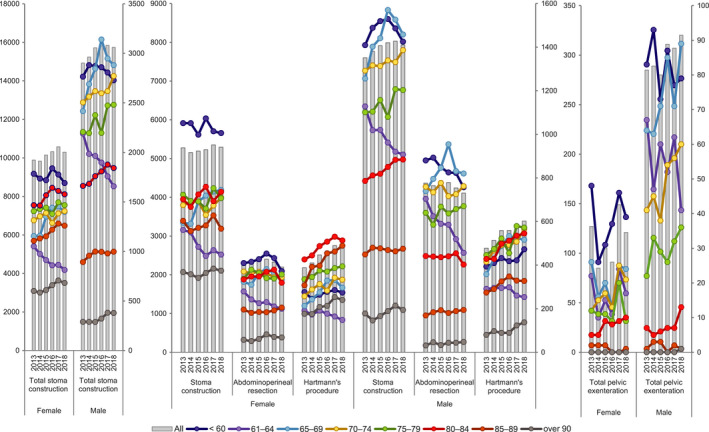
Annual changes of surgeries with stoma construction by sex and age group

During the study period, the number of gastrointestinal stoma construction cases per year increased gradually in both men and women, and the proportion of older patients increased every year. In a comparison of male and female patients, there were 93,271 men and 61,052 women. In patients aged 85 y and older, the number of women was 10,994 and there were 7769 males. The number of stoma construction procedures (except for APR, TPE, Hartmann's procedure) increased among men but remained about the same for women during each year of the study period. The number of APRs performed was about the same each year in both women and men, and the number of Hartmann's procedures increased in both sexes. The number of gastrointestinal stoma constructions has increased gradually in Japan, and the proportion of older patients requiring this procedure increased each year (Table 1, Figure 1).

**TABLE 1:** Annual changes in stoma construction by sex and age group

Procedure	Year	Sex	All	Age group (y)							
				< 60	61–64	65–69	70–74	75–79	80–84	85–89	over 90
Total	2013	Female, n	9886	1784	1052	1155	1314	1406	1467	1107	601
		Male, n	14 924	2764	2190	2416	2504	2207	1661	893	289
	2014	Female, n	9828	1735	975	1157	1354	1429	1463	1131	584
		Male, n	15 238	2880	1984	2689	2562	2194	1683	957	289
	2015	Female, n	10 144	1719	913	1352	1388	1442	1567	1153	610
		Male, n	15 720	2872	1964	2845	2618	2376	1759	998	288
	2016	Female, n	10 325	1839	860	1441	1287	1378	1642	1219	659
		Male, n	15 814	2859	1897	3140	2597	2196	1807	996	322
	2017	Female, n	10 576	1776	865	1456	1384	1496	1611	1281	707
		Male, n	15 840	2806	1758	2947	2618	2473	1877	980	381
	2018	Female, n	10 293	1690	813	1398	1406	1467	1577	1260	682
		Male, n	15 735	2728	1657	2881	2771	2480	1842	997	379
Stoma construction (except for APR, TPE, Hartmann's procedure)	2013	Female, n	5277	1051	561	594	675	723	702	603	368
		Male, n	7599	1409	1128	1256	1292	1101	786	449	178
	2014	Female, n	5155	1051	550	590	689	694	667	556	358
		Male, n	7766	1488	1019	1402	1316	1104	811	480	146
	2015	Female, n	5193	998	484	691	692	692	724	571	341
		Male, n	7912	1519	1020	1441	1313	1157	819	477	166
	2016	Female, n	5228	1073	442	719	630	659	759	582	364
		Male, n	7988	1529	962	1570	1339	1080	850	469	189
	2017	Female, n	5350	1014	468	737	673	753	693	628	384
		Male, n	8030	1486	920	1526	1331	1207	883	463	214
	2018	Female, n	5293	1006	448	745	710	707	736	567	374
		Male, n	7932	1425	907	1458	1386	1203	884	475	194
Abdominoperineal resection	2013	Female, n	2306	409	279	323	370	338	334	196	57
		Male, n	4363	880	704	739	759	640	441	169	31
	2014	Female, n	2289	415	242	310	363	379	348	181	51
		Male, n	4300	893	624	795	734	585	439	184	46
	2015	Female, n	2346	427	225	367	369	364	348	185	61
		Male, n	4390	848	589	846	777	668	436	193	33
	2016	Female, n	2402	453	230	381	363	340	369	184	82
		Male, n	4381	825	585	954	716	637	440	180	44
	2017	Female, n	2328	432	210	363	344	338	379	192	70
		Male, n	4242	823	519	831	725	657	454	189	44
	2018	Female, n	2198	372	200	334	343	357	319	205	68
		Male, n	4108	757	456	820	762	670	402	194	47
Total pelvic exenteration	2013	Female, n	127	48	22	26	12	12	5	2	0
		Male, n	285	83	67	64	41	22	7	1	0
	2014	Female, n	85	26	10	16	15	11	5	2	0
		Male, n	289	93	47	63	45	33	5	3	0
	2015	Female, n	105	31	15	20	17	11	9	2	0
		Male, n	280	73	60	71	38	29	6	3	0
	2016	Female, n	91	37	11	13	13	9	8	0	0
		Male, n	311	87	52	85	54	26	7	0	0
	2017	Female, n	149	46	24	25	25	20	9	0	0
		Male, n	307	77	62	71	56	32	7	2	0
	2018	Female, n	121	39	17	24	21	9	10	1	0
		Male, n	320	79	41	89	60	36	13	1	1
Hartmann's procedure	2013	Female, n	2181	276	191	213	257	335	426	307	176
		Male, n	2685	393	291	358	415	446	428	274	80
	2014	Female, n	2304	245	173	242	288	345	444	392	175
		Male, n	2890	409	295	430	467	474	428	290	97
	2015	Female, n	2510	264	191	275	313	375	487	397	208
		Male, n	3140	432	295	488	491	522	498	325	89
	2016	Female, n	2610	277	177	329	283	370	507	454	213
		Male, n	3145	418	298	533	492	455	512	348	89
	2017	Female, n	2754	285	164	332	342	386	530	462	253
		Male, n	3279	422	260	524	507	579	535	329	123
	2018	Female, n	2687	274	148	296	333	394	513	489	240
		Male, n	3387	472	253	516	565	572	545	327	137

Note

Total is the sum of stoma construction only or bowel resection with stoma construction, abdominoperineal resection, total pelvic exenteration, and Hartmann's procedure.

Abbreviations: APR, abdominoperineal resection; TPE, total pelvic exenteration.

### 3.2 Annual changes in stoma construction and closure by sex and age group

**FIGURE 2:**
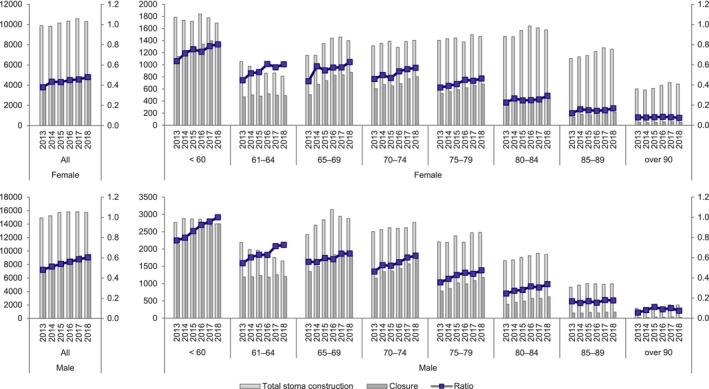
Annual changes of stoma construction and closure by sex and age group

In total, there were 77,910 cases of stoma closure during the study period, with 26,804 cases among women and 51,106 among men. In a comparison by sex, as with colostomy, there were more men than women under 85 y of age and more women than men over 85 y of age. The ratio of stoma closure to stoma construction by age group in each year increased among patients aged 79 y or younger. The ratio of stoma closure to stoma construction in those aged under 60 y increased each year, from 0.64 for women and 0.77 for men in 2013 to 0.80 for women and 1.00 for men in 2018 (Figure 2, Table S1).

### 3.3 Number of stoma and nonstoma constructions, according to surgical technique, by sex and age group

The number of total colectomies was 7788 (in 3182 women and 4606 men). Among them, 5090 cases (2073 women, 65.14%; 3017 men, 65.50%) were nonstoma construction and 2698 cases (1109 women, 1589 men) were stoma construction. Surgery with nonstoma construction in both sexes was performed in ~55% of patients under 60 y of age and 70%–75% in those over 70 y of age.

The total number of patients with proctocolectomy and ileoanal anastomosis was 2470 (924 women and 1546 men). Among them, 1018 cases (380 women and 638 men, 41.13% and 41.27%, respectively) were nonstoma construction and 1452 cases (544 women and 908 men) were stoma construction. As with total colectomy, surgery with nonstoma construction in both sexes was performed in ~40% of patients under 60 y of age and in 30%–50% of patients over 60 y of age. Although there was no difference between men and women, there were 1875 patients under the age of 60 y, accounting for 76% of the total.

The total number of LARs was 98 971 (34,555 women and 64,416 men).

**FIGURE 3:**
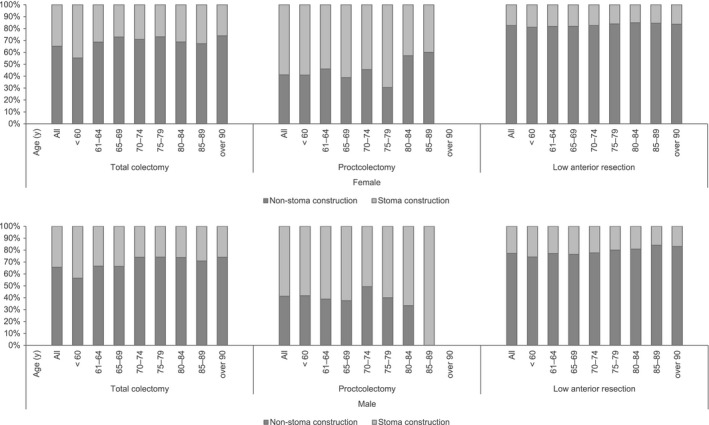
Number of stoma construction and no‐construction according to the three colorectum operative procedures by sex and age group

Although there was no difference by sex, 23,001 patients were under the age of 60 y, accounting for 76% of the total. Among them, 78,276 cases (women: 28 499 [82.47%], men: 49 777 [77.27%]) were nonstoma construction and 20,695 cases (6056 women, 14,639 men) were stoma construction. In a comparison by sex, the percentage of nonstoma construction was 82.47% in women and 77.27% in men (Figure 3, Table S2).

### 3.4 Patient characteristics with stoma and nonstoma construction according to surgical procedures for rectal cancer

**FIGURE 4:**
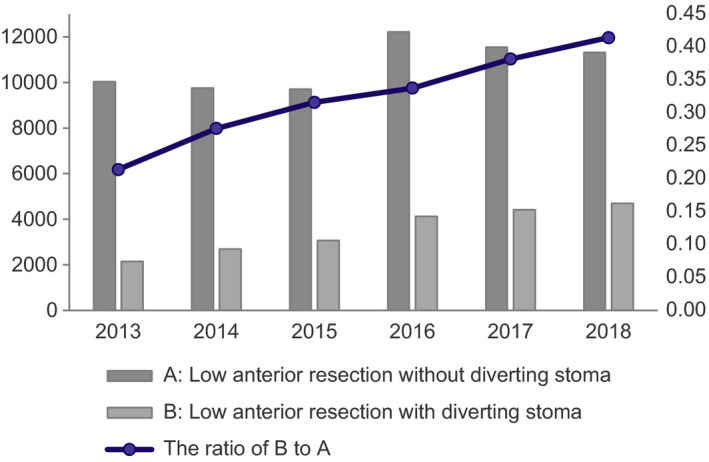
Number of low anterior resection with/without diverting stoma

Table 2 shows the characteristics of LAR with stoma construction, LAR without stoma construction (nonstoma construction), APR, Hartmann's procedure, and stoma construction without intestinal resection. In total, 21,122 patients had stoma construction and 64,572 had nonstoma construction in LAR. There was no significant difference in the characteristics of patients with and without stomas. The ratio of diverting stoma in LAR to LAR without stoma construction increased each year (Figure 4).

**TABLE 2:** Patient characteristics for stoma and nonstoma construction according to surgical procedures for rectal cancer (ICD‐10 code C20, malignant neoplasms of the rectum)

	Low anterior resection		Abdominoperineal resection	Hartmann's procedure	
	Without diverting stoma	With diverting stoma			Stoma construction without intestinal resection
	n = 64 572	n = 21 122	n = 1412	n = 977	n = 106
Age (y), median (IQR)	68 (61–75)	67 (60–74)	70 (63–77)	74 (66–81)	68 (61–75)
Sex					
Female, n (%)	22 811 (35.33)	6068 (28.73)	405 (28.68)	339 (34.70)	41 (38.68)
Male, n (%)	41 761 (64.67)	15 054 (71.27)	1007 (71.32)	638 (65.30)	65 (61.32)
Year					
2013, n (%)	10 030 (15.53)	2144 (10.15)	229 (16.22)	128 (13.10)	14 (13.21)
2014, n (%)	9748 (15.1)	2692 (12.75)	219 (15.51)	143 (14.64)	21 (19.81)
2015, n (%)	9707 (15.03)	3067 (14.52)	269 (19.05)	171 (17.50)	15 (14.15)
2016, n (%)	12 213 (18.91)	4122 (19.52)	232 (16.43)	170 (17.40)	18 (16.98)
2017, n (%)	11 552 (17.89)	4409 (20.87)	235 (16.64)	176 (18.01)	21 (19.81)
2018, n (%)	11 322 (17.53)	4688 (22.19)	228 (16.15)	189 (19.34)	17 (16.04)
Preoperative chemotherapy‐30					
Available, n (%)	1281 (1.98)	806 (3.82)	59 (4.18)	31 (3.17)	4 (3.77)
Not available, n (%)	63 291 (98.02)	20 316 (96.18)	1353 (95.82)	946 (96.83)	102 (96.23)
Preoperative chemotherapy‐90					
Available, n (%)	3653 (5.66)	3330 (15.77)	208 (14.73)	62 (6.35)	7 (6.60)
Not available, n (%)	60 919 (94.34)	17 792 (84.23)	1204 (85.27)	915 (93.65)	99 (93.4)
Radiation therapy‐90					
Available, n (%)	1292 (2.00)	1950 (9.23)	109 (7.72)	17 (1.74)	3 (2.83)
Not available, n (%)	63 280 (98.00)	19 172 (90.77)	1303 (92.28)	960 (98.26)	103 (97.17)
ASA‐PS					
ASA‐PS1, n (%)	19 111 (29.6)	4935 (23.36)	331 (23.44)	86 (8.80)	23 (21.70)
ASA‐PS2, n (%)	39 261 (60.8)	13 946 (66.03)	889 (62.96)	517 (52.92)	55 (51.89)
ASA‐PS3, n (%)	6067 (9.40)	2175 (10.3)	172 (12.18)	316 (32.34)	25 (23.58)
ASA‐PS4, n (%)	108 (0.17)	50 (0.24)	18 (1.27)	40 (4.09)	1 (0.94)
ASA‐PS5, n (%)	25 (0.04)	16 (0.08)	2 (0.14)	18 (1.84)	2 (1.89)
Tumor stage					
T0 or Tis orT1, n (%)	10 420 (16.14)	3417 (16.18)	71 (5.03)	25 (2.56)	1 (0.94)
T2, n (%)	11 867 (18.38)	4299 (20.35)	239 (16.93)	62 (6.35)	4 (3.77)
T3, (%)	32 488 (50.31)	10 424 (49.35)	816 (57.79)	495 (50.67)	20 (18.87)
T4a, n (%)	7910 (12.25)	2078 (9.84)	146 (10.34)	254 (26.00)	19 (17.92)
T4b, n (%)	1713 (2.65)	816 (3.86)	128 (9.07)	126 (12.9)	39 (36.79)
TX, n (%)	174 (0.27)	88 (0.42)	12 (0.85)	15 (1.54)	23 (21.70)
Node stage					
N0, n (%)	36 826 (57.03)	12 216 (57.84)	677 (47.95)	394 (40.33)	12 (11.32)
N1a or N1b or N1c, n (%)	18 069 (27.98)	5444 (25.77)	412 (29.18)	305 (31.22)	18 (16.98)
N2a or N2b, n (%)	9427 (14.6)	3348 (15.85)	305 (21.6)	207 (21.19)	30 (28.30)
NX, n (%)	250 (0.39)	114 (0.54)	18 (1.27)	71 (7.27)	46 (43.40)
Metastasis stage					
M0, n (%)	59 060 (91.46)	19 148 (90.65)	1105 (78.26)	698 (71.44)	52 (49.06)
M1, n (%)	5512 (8.54)	1974 (9.35)	307 (21.74)	279 (28.56)	54 (50.94)
Type of surgery					
Elective surgery, n (%)	64 147 (99.34)	20 799 (98.47)	1345 (95.25)	610 (62.44)	84 (79.25)
Emergency surgery, n (%)	425 (0.66)	323 (1.53)	67 (4.75)	367 (37.56)	22 (20.75)
Approach					
Nonendoscopic surgery, n (%)	29 695 (45.99)	7355 (34.82)	834 (59.07)	756 (77.38)	89 (83.96)
Endoscopic surgery, n (%)	34 877 (54.01)	13 767 (65.18)	578 (40.93)	221 (22.62)	17 (16.04)
Resection margin					
R0, n (%)	60 458 (93.63)	19 745 (93.48)	1250 (88.53)	701 (71.75)	17 (16.04)
R1, n (%)	862 (1.33)	368 (1.74)	65 (4.60)	57 (5.83)	1 (0.94)
R2, n (%)	2846 (4.41)	855 (4.05)	80 (5.67)	178 (18.22)	75 (70.75)
RX, n (%)	406 (0.63)	154 (0.73)	17 (1.20)	41 (4.20)	13 (12.26)

Abbreviations: ASA‐PS, American Society of Anesthesiologists physical status; ICD‐10, International Classification of Diseases, Tenth Revision; IQR, interquartile range; Preoperative chemotherapy‐30, preoperative chemotherapy within 30 d before cancer surgery; Preoperative chemotherapy‐90, preoperative chemotherapy within 90 d before cancer surgery; Radiation therapy‐90, radiation therapy within 90 d before cancer surgery.

The rate of stage T4b was 2.65% for LAR without diverting stoma and 3.86% for diverting stoma. The APR rate was 9.07%, Hartmann's technique 12.9%, and stoma construction without bowel resection 36.79%. Hartmann's procedure was characterized by a higher percentage of ASA‐PS 4 and 5 (5.9%) compared with other procedures, with fewer than 1% for LAR and 1.41% for APR (Table 2).

### 3.5 Patient characteristics with malignant and benign tumors in LAR

The characteristics of each malignant neoplasm and benign tumor in LAR are shown in Table 3. There were 93,546 cases of malignant neoplasm, 39 cases of benign tumors, and 5386 cases were unknown. Among malignant neoplasm surgeries, 14.24% of patients in the stoma group and 5.34% in the nonstoma group received preoperative chemotherapy 30 d before surgery. The percentage of patients who received preoperative chemotherapy 90 d prior to surgery was 3.58% in the stoma group and 1.89% in the nonstoma group. The preoperative radiation therapy rate was 8.46% in the stoma group and 1.78% in the nonstoma group. Nonlaparoscopic surgery was performed in 33.69% of the stoma group and 45.94% of the nonstoma group. There were no differences in the other patient characteristics (Table 3).

**TABLE 3:** Patient characteristics regarding malignant and benign tumors in low anterior resection

	Low anterior resection		Low anterior resection	
	Malignant neoplasm		Benign tumor	
	stoma construction	nonstoma construction	stoma construction	nonstoma construction
	n = 19 181	n = 74 365	n = 13	n = 26
Age (y), mean (SD)	66.18 (11.11)	67.41 (11.33)	62.31 (13.33)	65.81 (10.22)
Sex				
Female, n (%)	5440 (28.36)	26 377 (35.47)	4 (30.77)	11 (42.31)
Male, n (%)	13 741 (71.64)	47 988 (64.53)	9 (69.23)	15 (57.69)
Year				
2013, n (%)	1906 (9.94)	11 491 (15.45)	0 (0)	5 (19.23)
2014, n (%)	2366 (12.34)	11 215 (15.08)	3 (23.08)	3 (11.54)
2015, n (%)	2741 (14.29)	11 143 (14.98)	1 (7.69)	3 (11.54)
2016, n (%)	3745 (19.52)	14 098 (18.96)	5 (38.46)	4 (15.38)
2017, n (%)	4109 (21.42)	13 379 (17.99)	3 (23.08)	7 (26.92)
2018, n (%)	4314 (22.49)	13 039 (17.53)	1 (7.69)	4 (15.38)
Diabetes mellitus				
Insulin therapies, n (%)	15 531 (80.97)	60 875 (81.86)	12 (92.31)	21 (80.77)
No treatment, n (%)	308 (1.61)	1468 (1.97)	0 (0)	0 (0)
Diet therapy, n (%)	2299 (11.99)	8559 (11.51)	1 (7.69)	5 (19.23)
Oral agents, n (%)	735 (3.83)	2362 (3.18)	0 (0)	0 (0)
Dyspnea	308 (1.61)	1101 (1.48)	0 (0)	0 (0)
Not available, n (%)	18 968 (98.89)	73 515 (98.86)	12 (92.31)	26 (100)
During moderate exertion, n (%)	180 (0.94)	763 (1.03)	1 (7.69)	0 (0)
At rest, n (%)	33 (0.17)	87 (0.12)	0 (0)	0 (0)
ADL‐30				
Independent, n (%)	18 592 (96.93)	71 754 (96.49)	13 (100)	26 (100)
Partial assistance	479 (2.5)	2247 (3.02)	0 (0)	0 (0)
Total assistance	110 (0.57)	364 (0.49)	0 (0)	0 (0)
ADL‐surgery				
Independent, n (%)	18 512 (96.51)	71 500 (96.15)	12 (92.31)	26 (100)
Partial assistance	535 (2.79)	2454 (3.3)	1 (7.69)	0 (0)
Total assistance	134 (0.7)	411 (0.55)	0 (0)	0 (0)
COPD				
Not available, n (%)	18 318 (95.5)	71 922 (96.71)	12 (92.31)	25 (96.15)
Available, n (%)	863 (4.5)	2443 (3.29)	1 (7.69)	1 (3.85)
Dialysis‐14				
Not available, n (%)	19 076 (99.45)	73 999 (99.51)	13 (100)	25 (96.15)
Available, n (%)	105 (0.55)	366 (0.49)	0 (0)	1 (3.85)
Multiple metastases				
Not available, n (%)	18 779 (97.9)	72 298 (97.22)	13 (100)	26 (100)
Available, n (%)	402 (2.1)	2067 (2.78)	0 (0)	0 (0)
Steroid therapy				
Not available, n (%)	18 987 (98.99)	73 841 (99.3)	12 (92.31)	24 (92.31)
Discontinuation 30 d before surgery, n (%)	24 (0.13)	80 (0.11)	0 (0)	1 (3.85)
Available, n (%)	170 (0.89)	444 (0.6)	1 (7.69)	1 (3.85)
ASA‐PS ASA‐PS				
ASA‐PS1, n (%)	4534 (23.64)	21 691 (29.17)	8 (61.54)	6 (23.08)
ASA‐PS2, n (%)	12 596 (65.67)	45 401 (61.05)	3 (23.08)	18 (69.23)
ASA‐PS3, n (%)	1988 (10.36)	7106 (9.56)	2 (15.38)	2 (7.69)
ASA‐PS4, n (%)	49 (0.26)	137 (0.18)	0 (0)	0 (0)
ASA‐PS5, n (%)	14 (0.07)	30 (0.04)	0 (0)	0 (0)
Type of surgery				
Elective surgery, n (%)	18 893 (98.5)	73 824 (99.27)	12 (92.31)	26 (100)
Emergency surgery, n (%)	288 (1.5)	541 (0.73)	1 (7.69)	0 (0)
Approach				
Nonendoscopic surgery, n (%)	6462 (33.69)	34 161 (45.94)	5 (38.46)	10 (38.46)
Endoscopic surgery, n (%)	12 719 (66.31)	40 204 (54.06)	8 (61.54)	16 (61.54)
Preoperative chemotherapy‐30				
Available, n (%)	2732 (14.24)	3968 (5.34)	0 (0)	2 (7.69)
Not available, n (%)	16 449 (85.76)	70 397 (94.66)	13 (100)	24 (92.31)
Preoperative chemotherapy‐90				
Available, n (%)	686 (3.58)	1408 (1.89)	0 (0)	0 (0)
Not available, n (%)	18 495 (96.42)	72 957 (98.11)	13 (100)	26 (100)
Radiation therapy‐90				
Available, n (%)	1623 (8.46)	1324 (1.78)	0 (0)	0 (0)
Not available, n (%)	17 558 (91.54)	73 041 (98.22)	13 (100)	26 (100)
Tumor stage				
T stage, T0, n (%)	127 (0.66)	303 (0.41)		
Tis, n (%)	259 (1.35)	1202 (1.62)		
T1, n (%)	2856 (14.92)	10 064 (13.54)		
T2, n (%)	3974 (20.77)	13 123 (17.66)		
T3, n (%)	9300 (48.6)	37 373 (50.3)		
T4a, n (%)	1908 (9.97)	9685 (13.03)		
T4b, n (%)	632 (3.3)	2321 (3.12)		
TX, n (%)	80 (0.42)	231 (0.31)		
Node stage				
N stage, N0, n (%)	11 247 (58.77)	42 080 (56.63)		
N1a, n (%)	2594 (13.56)	11 557 (15.55)		
N1b, n (%)	2179 (11.39)	9179 (12.35)		
N1c, n (%)	81 (0.42)	298 (0.4)		
N2a, n (%)	1767 (9.23)	6860 (9.23)		
N2b, n (%)	1185 (6.19)	3984 (5.36)		
NX, n (%)	83 (0.43)	344 (0.46)		
Metastasis stage				
M stage, M0, n (%)	17 584 (91.89)	67 656 (91.06)		
M1a, n (%)	1221 (6.38)	5036 (6.78)		
M1b, n (%)	308 (1.61)	1512 (2.03)		
M1c, n (%)	23 (0.12)	98 (0.13)		
UICC stage				
Stage, 0, n (%)	252 (1.33)	1178 (1.6)		
Stage, 1, n (%)	5625 (29.72)	18 949 (25.68)		
Stage, 2, n (%)	4896 (25.87)	20 358 (27.59)		
Stage, 3, n (%)	6601 (34.88)	26 652 (36.12)		
Stage, 4, n (%)	1552 (8.2)	6646 (9.01)		

Abbreviations: ADL‐30, activities of daily living within 30 d before surgery; ADL‐surgery, activities of daily living just before surgery; COPD, chronic obstructive pulmonary disease; Dialysis‐14, dialysis within 14 d before surgery; Dyspnea, dyspnea within 30 d before surgery; Multiple metastases, highly advanced cancer with multiple metastases just before surgery; Preoperative chemotherapy‐30, preoperative chemotherapy within 30 d before cancer surgery; Preoperative chemotherapy‐90, preoperative chemotherapy within 90 d before cancer surgery; Radiation therapy‐90, radiation therapy within 90 d before cancer surgery; SA‐PS, American Society of Anesthesiologists physical status; Steroid therapy, long‐term corticosteroid therapy; UICC, Union for International Cancer Control.

## 4 DISCUSSION

In this study we found that the number of cases of stoma construction in Japan has been increasing slowly, and the number of these patients in their 70s and older has been increasing each year. This finding may be due to the fact that some older patients chose to have a stoma construction procedure because of the safety of perioperative management and defecation care^10^ or because they have difficulty with stoma closure owing to poor surgical tolerance. This may be true for stoma closure because the ratio of stoma closure to construction has increased in patients under 84 y of age but not in patients over 85 y of age. The high ratio of stoma closure to stoma construction in patients under 59 y of age can also be explained by the fact that surgery for inflammatory bowel disease is often combined with temporary stoma construction,^11^ resulting in a high incidence among younger patients.^12^

The total number of stoma construction cases was 24,810 in 2013, 25,066 in 2014, 25,864 in 2015, 26,139 in 2016, 26,416 in 2017, and 26,028 in 2018. However, the number of applications for physical disability certificates owing to rectal/bladder dysfunction with a registered permanent stoma is ~30,000 each year in Japan.^7^ Even though the results of this study reflect the sum of temporary and permanent stomas, the total was lower than the number of registrations for physically disability. When we looked at the presence or absence of stoma construction for the three techniques in total colectomy, proctocolectomy, and LAR, the rate of stoma construction was highest for low anterior resection, and stoma construction was performed in ~82% of all cases. Because all of these surgeries are combined with anastomosis of the intestine with preservation of the anus, we considered that this type of stoma construction is positioned as a diverting stoma. In the 1990s and 2000s, it was reported that temporary stoma placement significantly prevented suture failure in low anterior resection.^13^, ^14^, ^15^, ^16^, ^17^, ^18^ Later, in the 2010s, the impact of temporary stoma construction on rates of complication other than suture failure was also examined.^14^ Insurance coverage for stents beginning in 2012 has enabled preoperative decompression for colorectal cancer obstruction, decreased the rate of stoma construction before cancer chemotherapy, ^19^ and has reportedly prevented suture failure in transanal anal drains.^20^, ^21^ Whereas the indications for diverting stoma placement are diminishing, there are also reports of risks associated with the use of transanal drains.^18^, ^22^ Additionally, developments such as intraoperative flexible sigmoidoscopy have influenced the widespread use of intraoperative suture confirmation and restorative interventions,^23^ and the indications for diverting stoma construction to prevent suture failure and subsequent recurrence in these anus‐preserving surgeries are unclear among different institutions and surgeons.^24^ Furthermore, these complications and risks are different in robot‐assisted surgery.^25^, ^26^ Robot‐assisted surgery has also been reported to have a higher rate of stoma construction than non‐robot‐assisted surgery.^25^ In Japan, robotic‐assisted surgery in the lower rectum has been covered by the national health insurance since 2018. Therefore, future studies should consider robotic‐assisted surgery and other types of surgery. Thus, it is expected that the indications for diverting stoma will be transformed with the evolution of surgical instruments, equipment, and techniques.

In rectal cancer, APR, Hartmann's procedure, and stoma construction are characterized by stage progression, as compared with low anterior resection. In particular, Hartmann's procedure has a closure rate of 46%^27^ owing to bowel perforation or malignant obstruction as an emergency surgery, suggesting a background of a poor general condition.

Although there are reports that the presence or absence of concomitant stoma construction in LAR is related to age, low albumin, tumor size, distance from the anus, and rectal pressure,^26^ the results of the present study showed that the prevalence of preoperative chemotherapy and radiation therapy is a decision‐making factor for stoma construction specific to malignant disease.

Surgery for rectal cancer, a typical disease for which a gastrointestinal stoma is placed, can range from APR to LAR to preserve the anus and can require the placement of a permanent stoma, a temporary stoma, or no stoma.^13^

In particular, the rate of stoma construction following curative surgery for rectal cancer is decreasing owing to advances in anus‐preserving surgical equipment and techniques.^28^ However, the number of stomas is expected to increase in the future, given the increase in the number of patients with rectal cancer and surgeries.^6^, ^29^ The modest increase in the number of stomas constructed over the 5‐year study period may reflect these factors. However, treatment methods progress and change each year. For example, the number of stoma constructions is expected to decrease owing to progress in cancer treatment and the expansion of indications for treatment of gastrointestinal obstruction such as gastrointestinal stenting.^30^, ^31^, ^32^ Stoma construction as a treatment strategy prior to neoadjuvant chemotherapy for advanced colorectal cancer, palliative stoma,^33^ and stoma construction as a countermeasure for complications of other diseases and treatments are also increasing,^34^ because the period to resection surgery can be longer than that for stenting.^35^ Additionally, stoma construction surgeries are also performed for benign diseases, such as emergency surgery for colonic perforation.^36^, ^37^ Thus, it is necessary to consider that the purpose and indications for stoma construction will change and to look at the future trends. In Japan, where the proportion of older people is the highest in the world, the low ratio of stoma closure in the oldest patients in this study and the indications for stoma construction in older people (8) suggest that the number of gastrointestinal ostomates, especially in super‐aged populations, will continue to increase in the future.

### 4.1 Limitations

In this survey, the background for the construction of a gastrointestinal stoma could not be clarified because it was not linked to the name of the disease in the data source used. Additionally, multiple terms are used to refer to surgical procedures used to create a gastrointestinal stoma, such as “colostomy,” which includes enterocutaneous fistula for the purpose of creating an excretion route and enterocutaneous fistula for a route of nutrition injection. Because the purpose of this survey was to determine the route of excretion, we excluded those procedures that were performed in conjunction with esophageal surgery so as to exclude those performed for nutritional infusion.

## 5 CONCLUSION

The number of gastrointestinal stomas registered in the NCD during the study period was approximately 25,000 per year, with a moderate increase during 2013–2018. The number of stoma closures was 10,000–14,400 per year. The number of stoma closures has also increased. The ratio of concomitant stoma construction surgery was higher in older people, and the ratio of stoma closure was higher in younger patients. The purposes and surgical techniques of stoma construction are diverse and are expected to increase in Japan, which is a super‐aged society.

## DISCLOSURE

Funding information: This research was supported by the Japanese Society of Gastroenterological Surgery

Conflict of interest: Arata Takahashi, Hiroyuki Yamamoto, and Hiroaki Miyata are affiliated with the Department of Healthcare Quality Assessment at The University of Tokyo. The department is a social collaboration department supported by grants from the National Clinical Database, Johnson & Johnson KK, and Nipro Co. The remaining authors declare no conflicts of interest for this article.

Ethical Approval: The protocol for this research project was approved by a suitably constituted Ethics Committee of the institution and it conforms to the provisions of the Declaration of Helsinki. This study was approved by the hospital Ethics Review Board of the Osaka University Clinical Research Review Committee (approval number 18 292‐2).

Author contributions: A.T., Y.H. and M.Y. tabulated the data; Y.A. and M.F. drafted the article; Y.N., T.T., H.H., and T.K. proofread the content; and Y.K. and Y.K. gave final approval of the article. All authors have read and approved the final article.

## Supporting information

Table S1‐2

Supplementary Material
