# Supplementary material for: Survey Regarding Gastrointestinal Stoma Construction and Closure in Japan
Source: Ann Gastroenterol Surg. 2021 Nov 6;6(2):212–26. doi: 10.1002/ags3.12521 (PMC8889857; doi:10.1002/ags3.12521)
Supplement: Supplementary file 1 — Table S1‐2 [file AGS3-6-212-s001.docx]

Supporting table S1. Annual changes in stoma construction and closure by sex and age group.

| Year | Sex |  |  | Age group (years) | | | | | | | |
| --- | --- | --- | --- | --- | --- | --- | --- | --- | --- | --- | --- |
|  |  |  | All | < 60 | 61–64 | 65–69 | 70–74 | 75–79 | 80–84 | 85–89 | over 90 |
| 2013 | Female | Total of stoma construction, n | 9886 | 1784 | 1052 | 1155 | 1314 | 1406 | 1467 | 1107 | 601 |
|  |  | Stoma closure, n | 3757 | 1136 | 471 | 506 | 604 | 528 | 331 | 133 | 48 |
|  |  | Ratio | 0.38 | 0.64 | 0.45 | 0.44 | 0.46 | 0.38 | 0.23 | 0.12 | 0.08 |
|  | Male | Total of stoma construction, n | 14924 | 2764 | 2190 | 2416 | 2504 | 2207 | 1661 | 893 | 289 |
|  |  | Stoma closure, n | 7183 | 2129 | 1193 | 1349 | 1158 | 784 | 405 | 149 | 16 |
|  |  | Ratio | 0.48 | 0.77 | 0.54 | 0.56 | 0.46 | 0.36 | 0.24 | 0.17 | 0.06 |
| 2014 | Female | Total of stoma construction, n | 9828 | 1735 | 975 | 1157 | 1354 | 1429 | 1463 | 1131 | 584 |
|  |  | Stoma closure, n | 4269 | 1238 | 503 | 677 | 675 | 559 | 391 | 180 | 46 |
|  |  | Ratio | 0.43 | 0.71 | 0.52 | 0.59 | 0.50 | 0.39 | 0.27 | 0.16 | 0.08 |
|  | Male | Total of stoma construction, n | 15238 | 2880 | 1984 | 2689 | 2562 | 2194 | 1683 | 957 | 289 |
|  |  | Stoma closure, n | 7815 | 2298 | 1194 | 1495 | 1347 | 856 | 457 | 145 | 23 |
|  |  | Ratio | 0.51 | 0.80 | 0.60 | 0.56 | 0.53 | 0.39 | 0.27 | 0.15 | 0.08 |
| 2015 | Female | Total of stoma construction, n | 10144 | 1719 | 913 | 1352 | 1388 | 1442 | 1567 | 1153 | 610 |
|  |  | Stoma closure, n | 4367 | 1297 | 482 | 737 | 652 | 589 | 387 | 174 | 49 |
|  |  | Ratio | 0.43 | 0.75 | 0.53 | 0.55 | 0.47 | 0.41 | 0.25 | 0.15 | 0.08 |
|  | Male | Total of stoma construction, n | 15720 | 2872 | 1964 | 2845 | 2618 | 2376 | 1759 | 998 | 288 |
|  |  | Stoma closure, n | 8485 | 2480 | 1235 | 1695 | 1363 | 1019 | 493 | 168 | 32 |
|  |  | Ratio | 0.54 | 0.86 | 0.63 | 0.60 | 0.52 | 0.43 | 0.28 | 0.17 | 0.11 |
| 2016 | Female | Total of stoma construction, n | 10325 | 1839 | 860 | 1441 | 1287 | 1378 | 1642 | 1219 | 659 |
|  |  | Stoma closure, n | 4647 | 1343 | 523 | 826 | 690 | 622 | 411 | 176 | 56 |
|  |  | Ratio | 0.45 | 0.73 | 0.61 | 0.57 | 0.54 | 0.45 | 0.25 | 0.14 | 0.08 |
|  | Male | Total of stoma construction, n | 15814 | 2859 | 1897 | 3140 | 2597 | 2196 | 1807 | 996 | 322 |
|  |  | Stoma closure, n | 8852 | 2647 | 1189 | 1835 | 1440 | 990 | 570 | 153 | 28 |
|  |  | Ratio | 0.56 | 0.93 | 0.63 | 0.58 | 0.55 | 0.45 | 0.32 | 0.15 | 0.09 |
| 2017 | Female | Total of stoma construction, n | 10576 | 1776 | 865 | 1456 | 1384 | 1496 | 1611 | 1281 | 707 |
|  |  | Stoma closure, n | 4826 | 1396 | 498 | 836 | 771 | 662 | 414 | 192 | 57 |
|  |  | Ratio | 0.46 | 0.79 | 0.58 | 0.57 | 0.56 | 0.44 | 0.26 | 0.15 | 0.08 |
|  | Male | Total of stoma construction, n | 15840 | 2806 | 1758 | 2947 | 2618 | 2473 | 1877 | 980 | 381 |
|  |  | Stoma closure, n | 9274 | 2688 | 1256 | 1887 | 1573 | 1086 | 570 | 176 | 38 |
|  |  | Ratio | 0.59 | 0.96 | 0.71 | 0.64 | 0.60 | 0.44 | 0.30 | 0.18 | 0.10 |
| 2018 | Female | Total of stoma construction, n | 10293 | 1690 | 813 | 1398 | 1406 | 1467 | 1577 | 1260 | 682 |
|  |  | Stoma closure, n | 4938 | 1357 | 492 | 877 | 803 | 682 | 463 | 213 | 51 |
|  |  | Ratio | 0.48 | 0.80 | 0.61 | 0.63 | 0.57 | 0.46 | 0.29 | 0.17 | 0.07 |
|  | Male | Total of stoma construction, n | 15735 | 2728 | 1657 | 2881 | 2771 | 2480 | 1842 | 997 | 379 |
|  |  | Stoma closure, n | 9497 | 2730 | 1205 | 1847 | 1716 | 1175 | 621 | 175 | 28 |
|  |  | Ratio | 0.60 | 1.00 | 0.73 | 0.64 | 0.62 | 0.47 | 0.34 | 0.18 | 0.07 |

Total stoma constructions is stoma construction, abdominoperineal resection, total pelvic exenteration, and Hartmann’s procedure. Ratio is total stoma closure to total stoma construction.

Supporting table S2. Number of stoma and non-stoma constructions according to surgical procedure, by sex and age group.

|  |  |  |  | Age group (years) | | | | | | | |
| --- | --- | --- | --- | --- | --- | --- | --- | --- | --- | --- | --- |
|  |  |  | All | < 60 | 61–64 | 65–69 | 70–74 | 75–79 | 80–84 | 85–89 | over 90 |
| Total colectomy | | |  |  |  |  |  |  |  |  |  |
|  | Female | Construction, n | 1109 | 514 | 71 | 96 | 90 | 109 | 113 | 86 | 30 |
|  |  | Non-construction, n | 2073 | 635 | 155 | 258 | 219 | 295 | 249 | 177 | 85 |
|  |  | Proportion, % | 65.15 | 55.27 | 68.58 | 72.88 | 70.87 | 73.02 | 68.78 | 67.30 | 73.91 |
|  | Male | Construction, n | 1589 | 750 | 159 | 200 | 152 | 143 | 115 | 58 | 12 |
|  |  | Non-construction, n | 3017 | 970 | 315 | 393 | 432 | 409 | 323 | 141 | 34 |
|  |  | Proportion, % | 65.50 | 56.40 | 66.46 | 66.27 | 73.97 | 74.09 | 73.74 | 70.85 | 73.91 |
| Proctocolectomy | | |  |  |  |  |  |  |  |  |  |
|  | Female | Construction, n | 544 | 437 | 27 | 41 | 18 | 16 | 3 | 2 | 0 |
|  |  | Non-construction, n | 380 | 302 | 23 | 26 | 15 | 7 | 4 | 3 | 0 |
|  |  | Proportion, % | 41.13 | 40.87 | 46.00 | 38.81 | 45.45 | 30.43 | 57.14 | 60.00 | NA |
|  | Male | Construction, n | 908 | 662 | 88 | 85 | 36 | 24 | 12 | 1 | 0 |
|  |  | Non-construction, n | 638 | 474 | 56 | 51 | 35 | 16 | 6 | 0 | 0 |
|  |  | Proportion, % | 41.27 | 41.73 | 38.89 | 37.50 | 49.30 | 40.00 | 33.33 | 0.00 | NA |
| Low anterior resection | | |  |  |  |  |  |  |  |  |  |
|  | Female | Construction, n | 6056 | 1637 | 776 | 1059 | 944 | 781 | 523 | 265 | 71 |
|  |  | Non-construction, n | 28499 | 7019 | 3485 | 4782 | 4440 | 4048 | 2923 | 1437 | 365 |
|  |  | Proportion, % | 82.47 | 81.09 | 81.79 | 81.87 | 82.47 | 83.83 | 84.82 | 84.43 | 83.72 |
|  | Male | Construction, n | 14639 | 3705 | 2208 | 3157 | 2582 | 1727 | 940 | 267 | 53 |
|  |  | Non-construction, n | 49777 | 10640 | 7430 | 10237 | 8960 | 6897 | 3946 | 1409 | 258 |
|  |  | Proportion, % | 77.27 | 74.17 | 77.09 | 76.43 | 77.63 | 79.97 | 80.76 | 84.07 | 82.96 |

NA, not available.
