# Supplementary material for: Survey Regarding Gastrointestinal Stoma Construction and Closure in Japan
Source: Ann Gastroenterol Surg. 2021 Nov 6;6(2):212–26. doi: 10.1002/ags3.12521 (PMC8889857; doi:10.1002/ags3.12521)
Supplement: Supplementary file 2 — Supplementary Material [file AGS3-6-212-s002.docx]

**Supporting Information List**

Supporting table S1. Annual changes in stoma construction and closure by sex and age group.

Supporting table S2. Number of stoma and non-stoma constructions according to surgical procedure, by sex and age group.
